# Mechanisms Underlying Memory Consolidation by Adult-Born Neurons During Sleep

**DOI:** 10.3389/fncel.2020.594401

**Published:** 2020-11-26

**Authors:** Pablo Vergara, Masanori Sakaguchi

**Affiliations:** International Institute for Integrative Sleep Medicine (WPI-IIIS), University of Tsukuba, Tsukuba, Japan

**Keywords:** adult-neurogenesis, REM sleep, memory consolidation, theta oscillation, synaptic plasticity, hippocampus, optogenetics, calcium-imaging

## Abstract

The mammalian hippocampus generates new neurons that incorporate into existing neuronal networks throughout the lifespan, which bestows a unique form of cellular plasticity to the memory system. Recently, we found that hippocampal adult-born neurons (ABNs) that were active during learning reactivate during subsequent rapid eye movement (REM) sleep and provided causal evidence that ABN activity during REM sleep is necessary for memory consolidation. Here, we describe the potential underlying mechanisms by highlighting distinct characteristics of ABNs including decoupled firing from local oscillations and ability to undergo profound synaptic remodeling in response to experience. We further discuss whether ABNs constitute the conventional definition of engram cells by focusing on their active and passive roles in the memory system. This synthesis of evidence helps advance our thinking on the unique mechanisms by which ABNs contribute to memory consolidation.

## Introduction

The hippocampal dentate gyrus (DG) is one of a few regions in the mammalian brain where new neurons are generated throughout the lifespan (Altman, 1963). Adult neurogenesis is believed to benefit cognitive functions that aid survival (Kempermann, 2012) and is implicated in various aspects of memory processing, despite that it gives rise to a small number of neurons. This prompts the question of which characteristics distinguish adult-born neurons (ABNs) from developmentally born neurons, such as DG granular neurons (GNs). Indeed, 4- to 6-week-old ABNs possess several unique properties including increased synaptic plasticity and excitability (Schmidt-Hieber et al., 2004; Esposito, 2005; Ge et al., 2007; Gu et al., 2012) and reduced feedback inhibition (Temprana et al., 2015). Furthermore, ABNs can inhibit or excite GNs depending on whether ABN inputs are received from the lateral or medial entorhinal cortex, respectively (Luna et al., 2019). Thus, these properties of ABNs may bestow unique characteristics to the mammalian memory system.

**Figure 1:**
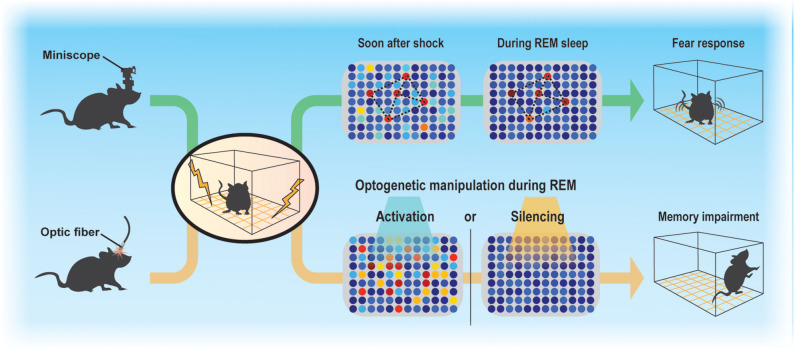
Sparse activity of ABNs during REM sleep is necessary for memory consolidation. Contextual fear conditioning recruits a subset of ABNs that reactivate in subsequent REM sleep. Disruption of ABN activity during REM sleep by optogenetic activation or silencing impairs memory consolidation.

Sleep regulates the magnitude of adult neurogenesis (Mirescu et al., 2006), which should thereby affect memory. However, whether and how adult neurogenesis affects memory consolidation during sleep is not well-understood, in part because examining the function of ABNs in specific sleep stages is technically challenging. Despite these difficulties, advances in Ca^2+^ imaging techniques along with the development of miniaturized fluorescent microscopes (Ghosh et al., 2011) and optogenetic approaches allowed us to demonstrate that even a small population of ABNs is necessary for memory consolidation during sleep (Figure 1) (Kumar et al., 2020). By analyzing the activity of ABNs in sleeping mice during memory consolidation, we found that, overall, ABNs become less active during rapid-eye movement (REM) sleep after mice form a fear memory consisting of an association between a context and shock but not after they are exposed to context or shock alone. Recently, Sorrells et al. (2018) cast doubt on the function of human adult neurogenesis considering its scarcity. However, we found that even the sparse activity of ABNs during REM sleep is necessary for memory consolidation, at least in mice, as both optogenetic activation and inhibition cause memory impairment. This could be because reactivation of a specific ABN population (e.g., a subset induced by learning) is required for memory consolidation during REM sleep. Indeed, optogenetic activation of a small fraction of non-specific hippocampal neurons can disrupt contextual memory retrieval (Iwasaki and Ikegaya, 2020). Another possibility is that specific temporal dynamics of ABN activity during REM sleep (e.g., spike timing to theta phase) are required for memory consolidation. Random optogenetic stimulation would disrupt both possible mechanisms.

In this review, we propose several mechanisms by which ABNs could mediate memory consolidation, particularly during REM sleep, placing special emphasis on the functional correlation of ABN activity with oscillatory dynamics in DG and CA3 circuits. We also discuss whether ABNs function in accordance with the canonical conception of engram cells.

## Contribution of Local Hippocampal Rhythms to Memory Consolidation

**Figure 2:**
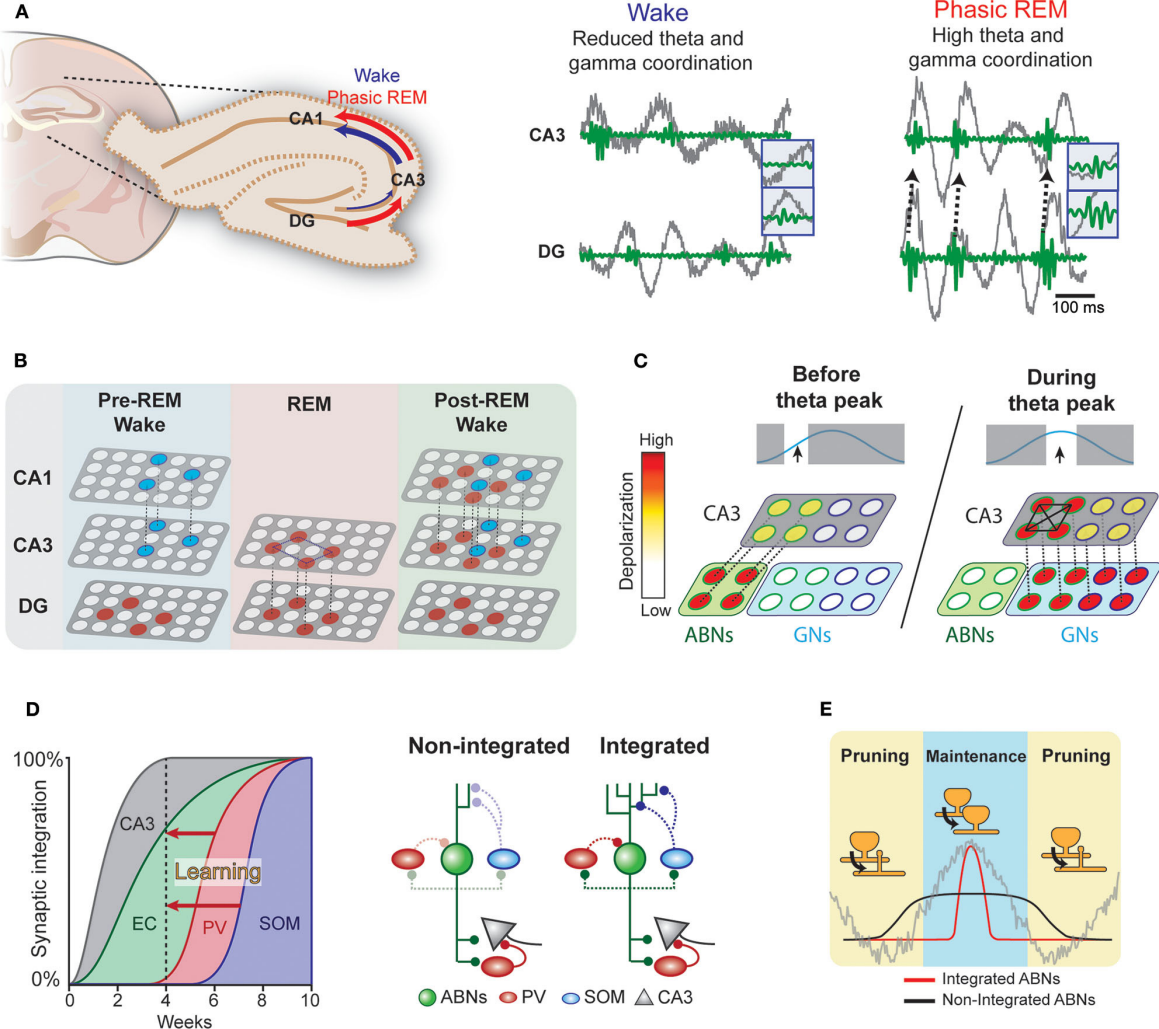
Hypothetical interplay between ABNs and local brain rhythms. **(A)** Left: Coherence in the DG-CA3-CA1 circuit during wakefulness and phasic REM sleep. Right: Coordinated activity in theta (gray) and gamma (green) ranges between the DG and CA3 (see Montgomery et al., 2008). **(B)** During wakefulness, neural ensembles stored in the CA3 may be transmitted to the CA1 and cortex (blue circles). During REM sleep, DG memory ensembles (red) may induce new auto-associative networks in the CA3. In subsequent wakefulness, modified CA3 networks may activate new neural ensembles in the CA1. **(C)** Enhanced intrinsic excitability and weak coupling to inhibitory circuits would allow a subset of ABNs to fire in response to weak perforant path input occurring before maximum theta excitability. These ABNs may depolarize CA3 pyramidal neurons and activate inhibitory circuits. During the peak of theta excitability, CA3 neurons that were depolarized by ABNs may be preferentially activated. The synchronized discharge of this specific CA3 population along with GNs may induce remodeling of CA3 recurrent connections. **(D)** Synaptic integration of ABNs into different circuits. Inhibitory inputs onto ABNs mature 8 weeks after mitosis. Learning could accelerate ABN integration into inhibitory circuits. **(E)** The activity of integrated but not non-integrated ABNs may couple to local oscillations. During subsequent REM sleep, synapses of oscillation-coupled but not -decoupled ABNs may be maintained.

During wakefulness and REM sleep, the hippocampus exhibits prominent theta oscillations (4–8 Hz), which may serve as a temporal reference for the encoding, processing, and decoding of memory traces. For example, consistent with the idea that theta rhythm may synchronize pre- and postsynaptic activity at entorhinal-DG synapses to induce long-term potentiation (LTP) following Hebbian rules (Levy and Steward, 1983), experimental observations show that theta phase-locked GN activity induces LTP in these synapses (Orr et al., 2001). Furthermore, coordinated activity in theta and gamma (30–100 Hz) ranges within the hippocampus may also be important for information transfer (Hanslmayr et al., 2016). Coherence between the DG and CA3 in the theta and gamma ranges is greater in REM sleep than in wakefulness (Montgomery et al., 2008) (Figure 2A). Therefore, theta rhythm in REM sleep may open a window for DG neurons to modulate CA3 circuits. Indeed, coordinated discharge of DG mossy fibers and CA3 neurons induce LTP in CA3 recurrent fibers (Kobayashi and Poo, 2004). The CA3 recurrent circuit is proposed to operate as an auto-associative network, allowing memory storage and later retrieval by partially reactivated neural ensembles (Rolls, 2007). REM sleep may facilitate modifications of this recurrent CA3 network via DG activity (Figure 2B). Because coordination between the CA3 and CA1 is higher during wakefulness, CA3 ensembles modified during REM sleep could trigger new CA1 ensemble activity during subsequent wakefulness.

## Interplay of ABNs With Local Hippocampal Rhythms

The influence of young ABNs on CA3 and DG coordinated activity may differ from that of GNs. Young ABNs functionally connect to both excitatory and inhibitory circuits in the CA3 to a similar extent as GNs (Temprana et al., 2015). Yet, ABNs receive weaker inhibitory inputs (Temprana et al., 2015; Groisman et al., 2020), which may prevent their spiking and synchronization with local theta (Amilhon et al., 2015) and gamma (Csicsvari et al., 2003; Bartos et al., 2007) oscillations. This also makes the spiking of ABNs less time-locked to perforant path inputs than GNs (Pardi et al., 2015). In addition, ABNs display greater membrane resistance than GNs (Esposito, 2005), allowing them to discharge in response to weaker entorhinal inputs. These properties may bestow ABNs with distinctive firing dynamics in relation to local oscillations. We speculate that these firing dynamics allow ABNs to (1) promote pattern separation by activating inhibitory circuits in the CA3 before GNs and/or (2) prime a subset of CA3 neurons to form a new memory representation (Rangel et al., 2013).

Several studies indicate that ABNs are necessary (Clelland et al., 2009) and sufficient (Sahay et al., 2011) for context discrimination. However, the exact underlying mechanism is unknown. At the circuit level, context discrimination is believed to occur via the decorrelation of neural activity patterns representing similar but different contexts, a process known as pattern separation (Hainmueller and Bartos, 2020). Pattern separation relies on the activation of inhibitory circuits and, as a consequence, sparse reactivation of neural ensembles (Cayco-Gajic and Silver, 2019). Within a theta cycle, ABNs may activate inhibitory circuits before GNs (Rangel et al., 2013), as ABNs would respond to inputs at a time when GN activity is highly inhibited. Moreover, the increased excitability of ABNs (Esposito, 2005) may make them responsive to weak entorhinal inputs, which would then drive CA3 inhibitory circuits. This may help establish non-overlapping memory traces in the CA3, which reduces memory interference.

ABNs may also interact with GNs to regulate recurrent circuits in the CA3. Coordinated spiking activity between the DG and CA3 may modify the functional connectivity of the CA3 recurrent circuit (Kobayashi and Poo, 2004). This coordinated activity is prominent during REM sleep (Montgomery et al., 2008) and most likely driven by GNs due to their stronger coupling with theta rhythm. ABNs with weak coupling to local oscillations due to their reduced synaptic inhibition and high intrinsic excitability may fire before GNs within a theta cycle. These preceding ABN spikes may prime a subset of CA3 neurons to be activated by subsequent synchronized GNs (i.e., a “priming” effect, Figure 2C) (Rangel et al., 2013). A single spike from a GN is unlikely to discharge a CA3 pyramidal neuron; rather, several spikes are needed (Henze et al., 2002). Thus, inputs from ABNs arriving slightly before those from GNs may provide initial depolarization that primes a subset of CA3 neurons to activate in response to subsequent theta-synchronized GN spikes. In contrast to unsynchronized ABN inputs, subsequent synchronized GNs inputs may enable synaptic adjustments in this specific CA3 subset (Kobayashi and Poo, 2004).

## Role of REM Sleep in ABN Synaptic Plasticity

The ongoing maturation of ABNs may support the segregation of memories over time (Aimone et al., 2006). That is, each cohort of ABNs may incorporate into different entorhinal and CA3 circuits when they have a high degree of plasticity. As each cohort matures, its plasticity decreases, making it less susceptible to retroactive interference. Thus, each cohort of ABNs may store a different memory. This model operates on a time scale of weeks, corresponding to the temporal dynamics of ABN maturation. Yet, the mechanisms by which the hippocampus circumvents retroactive interference over shorter time scales remains elusive. We speculate that fear learning enhances the integration of a subset of ABNs (Figure 2D). The enhanced synaptic plasticity of ABNs (Schmidt-Hieber et al., 2004; Ge et al., 2007; Gu et al., 2012) suggests that ABN synapses are more susceptible to experience-dependent modulation than GN synapses. Indeed, previous studies show that fear learning triggers profound modifications of ABN excitatory synapses within a few hours (Petsophonsakul et al., 2017; Kumar et al., 2020), and novelty exposure accelerates the integration of ABNs into both excitatory (Alvarez et al., 2016; Trinchero et al., 2019) and inhibitory circuits (Groisman et al., 2020). These findings suggest that experience influences the temporal dynamics of ABN synaptic maturation. Indeed, enhanced coupling of ABNs to inhibitory circuits may explain their overall decreased activity during REM sleep when fear memory is consolidated (Kumar et al., 2020). For instance, ABNs may establish a new input pathway from the hilus to somatostatin interneurons, which are activated by cholinergic binding to their M1 muscarine receptors (Raza et al., 2017). Therefore, increased cholinergic activity during REM sleep could induce sparse ABN activity. Because the subset of ABNs active during REM sleep largely overlaps with those active during learning, we hypothesize that the accelerated synaptic maturation of ABNs is input-specific. This early acquisition of a mature synaptic phenotype in a subset of ABNs would circumvent retroactive interference across shorter time scales.

The integration of a subset of ABNs into inhibitory circuits in response to learning may have several consequences for their synaptic processing during sleep (Figure 2E). Indeed, the prediction that ABNs fire decoupled from local oscillations arises from the fact that 4-week-old ABNs are weakly connected to local inhibitory circuits. If a subset of ABNs integrates with inhibitory circuits, the spikes of integrated but not non-integrated ABNs would couple to local hippocampal rhythms during REM sleep. Because DG neuron activity synchronized with local theta rhythm facilitates LTP of synapses with perforant path input (Orr et al., 2001), REM theta rhythm may selectively strengthen the synapses of integrated ABNs. Interestingly, we found that silencing ABN activity during REM sleep elongates their spine neck length (Kumar et al., 2020), a phenomenon associated with reduced synaptic strength (Araya et al., 2014) and desynchronization of synaptic input and postsynaptic neural activity (Tanaka et al., 2008). Collectively, these results suggest that ABN activity during REM sleep is necessary for maintaining the strength of their input synapses. Interestingly, a subset of learning-activated ABNs reactivates during REM sleep, suggesting that the spines of only some ABNs may strengthen during REM sleep. On the other hand, the spikes of non-integrated and oscillation-decoupled ABNs are likely to be decorrelated with input activity and thus prone to synaptic depression, consistently with the Hebbian rules of plasticity for GNs (Levy and Steward, 1983). Indeed, our optogenetic silencing may have decreased such coupling necessary for spine maintenance and the memory consolidation process. Interestingly, a recent study of the motor cortex indicates that REM sleep selectively strengthens and maintains the fraction of learning-induced new spines that are relevant for a motor task while at the same time prunes non-relevant synapses to facilitate subsequent memory acquisition (Li et al., 2017). Approaches with higher resolution, such as those used in cortical regions (Li et al., 2017), in combination with the tagging of activated spines (Hayashi-Takagi et al., 2015) may clarify the role of ABN synaptic plasticity during REM sleep.

## Are ABNs Engram Cells?

Fear memory engrams (i.e., traces) in the DG correspond to behaviorally relevant populations of neurons that are activated during both learning and memory retrieval (Liu et al., 2012; Denny et al., 2014). Although ABN activity is necessary for fear memory retrieval (Gu et al., 2012), we found little overlap between ABNs active during fear memory learning and retrieval (Kumar et al., 2020). This could be because the overall activity of ABNs relates to behavioral states but is not associated with contextual memory. For instance, place cells are not necessarily context-encoding cells in the CA1 (Tanaka et al., 2018). Moreover, even in GNs, which can hold an engram, there is little overlap between cells expressing immediate early genes during encoding and retrieval (Denny et al., 2014). This could be because immediate early gene-expressing GNs may segregate over time (Lamothe-molina et al., 2020). Therefore, a population of “active” cells may not simply represent a memory. Alternatively, a memory trace encoded by ABNs may decay upon the establishment of an engram in downstream circuits during REM sleep. This may allow ABNs to transiently encode new information while avoiding overlap with previously formed memory traces.

More work is needed to address how ABNs active during learning contribute to subsequent memory processing. A suitable approach for addressing the role of ABNs in establishing an engram would be to specifically manipulate ABNs that are active during learning, as previously done for GNs (Liu et al., 2012; Denny et al., 2014). Conversely, the specific activity pattern of memory-encoding ABNs may be distinguished from other ABNs by simultaneously imaging the activity of engram and non-engram ABNs and GNs in the DG, as previously done in the CA1 (Ghandour et al., 2019).

ABNs may also interact with engrams stored in GNs. Indeed, ABNs can directly inhibit or excite GNs depending on their inputs from the entorhinal cortex (Luna et al., 2019). This bidirectional control over GNs, and thus heightened ability to influence engrams, may only occur when ABNs are young. Across longer time scales, ABNs may also influence GN engrams by synaptic competition (McAvoy et al., 2016). Nonetheless, the mechanism by which ABNs interact with GNs during memory consolidation is largely unknown. Concurrent optogenetic manipulations of ABNs and imaging of GN activity during memory consolidation are required to solve this puzzle.

## Discussion

The exact mechanisms by which ABNs consolidate memory during REM sleep are unclear. Here, we proposed several mechanisms based on the known intrinsic properties and temporal activity dynamics of ABNs. However, there are many key questions to address in the future. (1) How does learning affect the functional connectivity of ABNs, particularly with inhibitory neurons? Electrophysiological recordings of ABNs during optogenetic manipulation of inhibitory neurons may shed light on this issue. (2) Are ABN spikes coupled to DG oscillations? Is this coupling affected by learning? Multielectrode recording and optogenetic tagging of ABNs may clarify their spiking dynamics, similar to techniques employed for mossy cells in the DG (Senzai and Buzsáki, 2017), although the sparse firing of ABNs could be a major obstacle. (3) How does ABN activity influence downstream circuits for memory consolidation? Imaging of CA3 neurons or GN activity combined with optogenetic manipulation of ABNs could clarify this issue. Such investigations would advance our understanding of the role of the unique plasticity mechanism of adult neurogenesis in the mammalian brain.

## Author Contributions

Conceptualization and Writing—Review & Editing: PV and MS. Visualization and Writing—Original Draft: PV. Funding Acquisition, Resources, and Supervision: MS. All authors discussed and approved the manuscript.

## Conflict of Interest

The authors declare that this study received funding from GSK Japan. The funder was not involved in the study design, collection, analysis, interpretation of data, the writing of this article or the decision to submit it for publication. The authors declare that the research was conducted in the absence of any commercial or financial relationships that could be construed as a potential conflict of interest.
